# Estimating the incidence of Venezuelan migration and other socioeconomic factors on urban growth in Colombia

**DOI:** 10.1371/journal.pone.0301552

**Published:** 2024-04-04

**Authors:** Pablo Blas Tupac Silva Barbosa, Andrés Peña-Galindo, Andrés Miguel Sampayo, Sebastian Londoño-Méndez, Ivan Enrique Contreras Cala, David Granada Donato, Jenny Rocío Beltrán Pérez, Alejandro Feged-Rivadeneira

**Affiliations:** 1 Department of Mathematics, Faculty of Sciences, Universidad Nacional, Bogotá, Colombia; 2 Faculty of Law and Political Sciences, Group of Political and Legal Studies, Universidad El Bosque, Bogotá, Colombia; 3 Faculty of International, Political, and Urban Studies, Universidad del Rosario, Bogotá, Colombia; 4 IMMAP, Bogotá, Colombia; University of Exeter, UNITED KINGDOM

## Abstract

This study investigates the intricate relationship between Venezuelan migration and urban growth in Colombia from 2018 to 2021. The study employs remote sensing data and social network metrics to uncover migration patterns and their impact on urban expansion. The methodology consists of three stages. Firstly, nighttime satellite imagery is used to analyze year-over-year urban growth in Colombia. Secondly, social network data estimates Venezuelan migration, overcoming challenges of underreporting and informal border crossings. Lastly, an econometric analysis explores the quantitative link between Venezuelan migration and urban growth, integrating socioeconomic variables to address endogeneity. The findings reveal the complex interplay of Venezuelan migration, socioeconomic factors, and urban growth. The study outlines remote sensing analysis, introducing the Anthropogenic Footprint Expansion Index (AFEI) to quantify urban growth. Facebook API data estimates migration trends and explores socioeconomic impacts on urban expansion. The analysis uncovers migration, poverty, aging, and urban population proportion as key factors affecting Colombia’s urban landscape. Furthermore, the research underscores how Venezuelan migration affected short-term urban expansion pre- and post-COVID-19. Migration had a notable effect before the pandemic, but this influence waned afterward. The study highlights migration’s short-term nature and emphasizes age demographics’ role in medium-term dynamics.

## Introduction

Venezuelan migration has emerged as a crucial concern for regional policy in Latin America and the Caribbean. This pervasive issue has spurred a wealth of research across a broad spectrum of fields, from the health implications [1] and mental health challenges [2] to educational impacts [3] in host countries. The capacity of the Western Hemisphere to manage the Venezuelan migration crisis has been critically examined [4], alongside the perceptions of criminality [5] and labor market effects [6] associated with the crisis. The figures from the International Organization for Migration (IOM) of the United Nations (UN) underline the prevalence of this phenomenon; by June 2021, approximately 5.6 million Venezuelans had fled their country, with about 4.6 million—85%—seeking refuge within the region, predominantly in Colombia, Peru, Chile, Ecuador, and Brazil. The IOM characterizes the ongoing Venezuelan situation as one of the most substantial displacement and migration crises globally.

With its extensive shared border, Colombia has become the principal sanctuary for Venezuelan migrants [7]. This influx has intensified the scholarly focus on Bogotá’s policy responses to the migration influx. Responses have ranged from militaristic [8] to critical poststructural approaches [9, 10]. The International Labor Organization (ILO), in concert with the United Nations Development Program (UNDP), has proposed strategies to uphold the labor rights of Venezuelan migrants in Colombia [11]. Despite the pressing need for empirical data to craft effective public policies, a lacuna exists in research exploring the impact of Venezuelan migration on Colombia’s urban expansion. Some studies do touch upon the socioeconomic aspects of urban growth and migration. For instance, some of them [12] delves into migrants’ motivations for leaving Venezuela, uncovering primarily economic drivers such as inflation and food scarcity. Migrants often settle in commercial zones and opt for flexible housing arrangements. Another study [13] presents insights on the settlement patterns of migrants, driven by affordable housing, economic prospects, and familial networks, albeit often in marginalized neighborhoods.

A geospatial perspective [14], utilizes satellite imagery and georeferencing to understand migrant urban integration and needs. Yet, publications specifically addressing urban growth in Colombia due to Venezuelan migration remain scarce, with most research concentrating on migrant rights [1, 15–18]. Internationally, studies on urban growth resulting from migration are similarly limited. Other research [19] compares the impacts of internal and international migration on urban growth in the Global South, suggesting a more pronounced effect from the latter. A different study [20] investigated external aid’s influence on reducing international migration, finding support for this hypothesis. Alternative research [21] explores the cyclical relationship between internal and international migration in Asia, a cycle further dissected in another study [22] to outline urban regions’ characteristics within this process. Other inquiries [23, 24] focus on urban growth and migration under severe economic or conflict-related conditions.

Research on internal migration in Colombia underscores the need for specialized policies to aid displaced individuals and curb the exacerbation of poverty due to migration [25–28]. These studies connect displacement with the Colombian armed conflict, showing a decline in well-being and highlighting the challenges of return programs. They introduce an index to measure the actual enjoyment of rights, with displaced families averaging at 60%, below that of impoverished households. Panel analysis from 2007 to 2008 reflects a regression in rights enjoyment among these families. Additional studies [29, 30] have probed urban transformation due to migration, indicating a positive correlation with forced migration due to conflict and a negative one with interregional displacement, revealing various anthropogenic changes over time and the challenges faced by internally displaced persons (IDPs) in urban settings.

In light of the extensive Venezuelan migration and its potential impact on Colombian urban growth, this article aims to dissect this underexplored dynamic, seeking to provide pertinent data for policy formulation that benefits migrants and host communities alike. The study traces the pre-pandemic effect of migration on urban expansion, particularly between 2018 and 2019. Although this influence persisted through the pandemic, its magnitude waned. Conversely, demographic changes and multidimensional poverty became more significant urban growth factors. Spatial autocorrelation within the data underpins the application of spatial econometric models utilized in this analysis.

To evaluate the hypothesis of Venezuelan migration’s pronounced effect on urban expansion, econometric analyses segmented the national territory by proximity to Colombian-Venezuelan border crossings. The results indicated that transportation costs do not critically influence Venezuelan migrants’ settlement decisions. Instead, migration’s impact is more significant in economically developed municipalities, aligning with Venezuelan migrants’ tendencies to integrate into economically vibrant communities.

Future research should investigate Venezuelan migration’s indirect and long-term effects on Colombian urban growth, including labor market and economic structural influences on cities. These studies should also validate this research’s findings with various datasets and consider additional variables like internal municipal migration and rural-urban dynamics, contributing to a more nuanced understanding of migration’s role in urban growth within the Colombian context.

## Remote sensing and social network connections data

This paper analyzes the short-term relationship between the migration dynamics of Venezuelans and the growth of urban areas in Colombia during the period 2018-2021. This period is of particular interest for two reasons: the first is that it coincides with a massive increase in the number of Venezuelan migrants in the country: according to National Administrative Department of Statistics [31] and the data obtained from Large Integrated Household Survey, statistical operation carried out by the National Administrative Department of Statistics (DANE), there has been a sustained growth in the total number of Venezuelan migrants between 2014 and 2020. The average annual variation of this population has been 80.6%, with the highest growth recorded between 2017 and 2018 at 167.5%. This period marked a significant intensification of the migratory phenomenon. As a result, by 2020, the total number of Venezuelan migrants reached 2.26 million people [31]; the second reason for the selection of the period of analysis is that it allows for an examination of migration dynamics on municipalities in the country before and after the COVID-19 pandemic. The wide availability and quality of geospatial data facilitates the study of the relationship between socio-economic phenomena, such as migration, and anthropogenic expansion, especially in relation to their impact on the physical environment and for diagnosing urban growth dynamics. This approach has been used in previous related research to study the effect of socio-economic phenomena on urban growth both in Colombia [30] and in several other countries [32–38].

The analysis of migratory dynamics presents many complexities, including those derived from the lack of robust statistics on the geographical location of migrants and their evolution over time. Although migration authorities in Colombia have formal records on the total number of Venezuelan migrants at the national and departmental level [39], there is no official information on the distribution of these migrants at the municipal level, nor a periodic uniform collection of this data. This situation is exacerbated by underreporting due to migratory flows through over 200 informal crossings along the 2,219-kilometer land border between Colombia and Venezuela [40].

Given this information gap, this study proposes the use of data on the scope of social networks as a proxy to approximate Venezuelan migration dynamics at the municipal level. This approach is an added value of the present research, as to our knowledge, there are no precedents in the literature regarding the study of the relationship between municipal-level migratory flow, approximated using data from social media reach, and its relation with urban growth in Colombia. Although this strategy may have limitations and endogeneity concerns (see [41]), it is seen as a suitable and statistically rigorous approach, in the absence of official records, to monitor migratory flows. Precedents for this approach can be found globally [42], in the United States [43], and in Europe [41].

## Study aims and objectives

In this academic article, we present a comprehensive methodology that leverages existing data and geospatial platforms to enhance our understanding of the complexities of the articulation of Venezuelan migration towards Colombia, which affect economic, demographic, social dynamics, among others, with the displacement of individuals towards urban areas. Our focus is on the unique context of Colombia, characterized by ongoing armed conflict and dynamic urbanization processes. Our primary goal is to examine the intricate relationship and dynamics between migration from Venezuela and land use and cover change, as well as other anthropogenic and socioeconomic transformations. With Colombia as our case study, our specific objectives revolve around employing remote sensing techniques to quantify nationwide anthropogenic changes spanning the period from 2018 to 2021, which coincides with a socio-politically significant phase of Venezuelan migration to Colombia. Subsequently, we delve into the discussion of how migration and socioeconomic factors, such as multidimensional poverty or the demographic structure of the population, can influence various aspects such as land use planning, public health systems, and ecological transformations. By adopting such an approach, we aim to enhance our comprehension of the political, demographic, and economic factors that drive anthropogenic alterations in land cover, as well as their influence on migration patterns and demographic shifts in developing nations.

## Materials and methods

The proposed methodology for this study encompasses three key stages. In the first stage, we measure year-over-year urban growth of the populated centers in Colombia for the period 2018-2021. In order to achieve this objective, we employed data from nighttime satellite imagery as a primary source, in consistency with previous research studies that have addressed similar objectives and have demonstrated the effectiveness of nighttime data in assessing urban expansion dynamics [30], which were cited in previous sections of this paper. In the second key stage, based on data on the reach of social networks, we estimate the number of Venezuelan migrants and their annual evolution in each municipality of Colombia. Finally, through different econometric specifications, we analyze the quantitative relationship between the two exposed phenomena, considering not only Venezuelan migration, but a complete set of economic and social variables that can explain the urban expansion and avoid problems of endogeneity in the econometric proposed model. The conceptual approach and the statistical treatment in each of the three stages of the proposed methodology are described below.

## Remote sensing analysis of nighttime lights data

In order to approximate the dynamics of urban expansion at the municipal level in the country, we identify those regions where the luminosity intensity (and consequently, the urban footprint) has significantly increased during the analyzed period. To achieve this objective, a significant data source was utilized: nighttime satellite images captured by the Earth Observation Group (EOG), which is part of the National Geophysical Data Center (NGDC) of the United States National Oceanic and Atmospheric Administration (NOAA). These images, openly accessible on the cloud-based geomatics Google Earth Engine platform, are sourced from Suomi National Polar Partnership satellite (NPP), provide crucial information on Average Day/Night Band (DNB) radiance values measured in nanoWatts/cm^2^/sr (band “avg_rad”) from the visible Infrared Imaging Radiometer Suite (VIIRS) sensor, which has a spatial resolution of 750 meters and 22 bands with a total spectral range of 4-12.5*μ*m. The DNB band has a wavelength range of 0.5-0.9*μ*m and 14 bits spectral resolution.

For each municipality *i*, the Anthropogenic Footprint Expansion Index from the year *t* to the year *t* + *k*, namely, *AFEI*^*i*^_[*t*,*t*+*k*]_ (Eq 1) was calculated as the ratio between variables *INRA*^*i*^_[*t*,*t*+*k*]_ and *TMA*^*i*^. On the one hand, *INRA*^*i*^_[*t*,*t*+*k*]_ corresponds the area of the municipality *i* that showed a significant increase in nighttime radiation in year *t*+ *k* with respect to year *t*. On the other hand, *TMA*^*i*^ represents the total area of the municipality *i*. We define *AFEI*^*i*^_[*t*,*t*+*k*]_ as:

$$\begin{array}{c} {AFEI^{i}}_{\left[t,t+k\right]}=\frac{{INRA^{i}}_{\left[t,t+k\right]}}{TMA^{i}}. \end{array}$$ (1)

This research proposes the study of the dynamics of urban expansion in the short term. Four periods of analysis are considered: *T*_1_ = [2018, 2019], *T*_2_ = [2019, 2020], *T*_3_ = [2020, 2021] and, finally, the entire period *T*_4_ = [2018, 2021].

**Fig 1:**
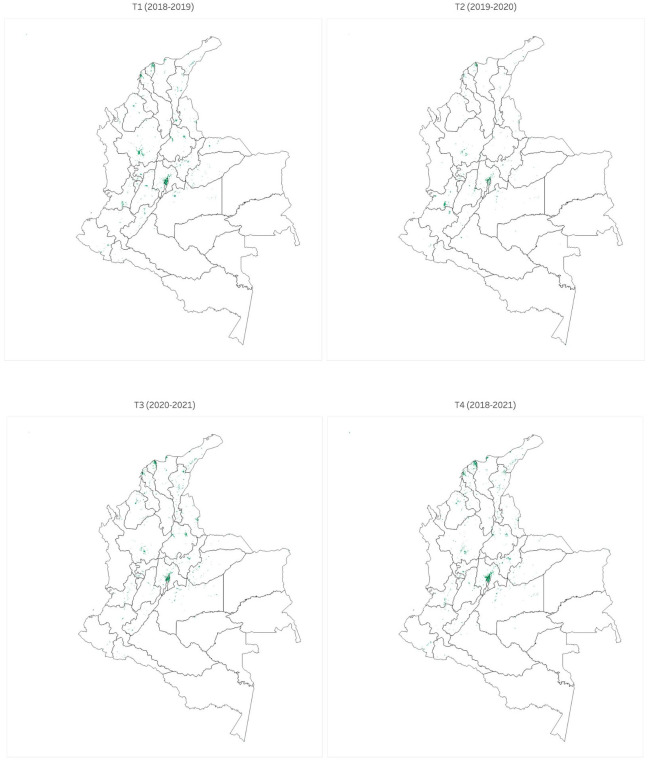
Areas of the national territory that experienced a significant increase in the radiation of nocturnal lights for periods *T*_1_ (i.e. 2018-2019) to *T*_4_ (i.e. 2018-2021).

The objective of constructing the index *AFEI* is to identify those municipalities that have experienced a significant increase in their urban area, approximating this variable by the nighttime light footprint. This, in turn, aims to establish the relationship between this phenomenon and the presence of Venezuelan migrants in the municipalities of the country. In Fig 1, the regions of the country that experienced a significant increase in urban area are illustrated, according to the *AFEI* index.

## An approach to the migratory dynamics of Venezuelans at the municipal level in Colombia

Data on connections among Venezuelan refugees and migrants in Latin America are obtained using Facebook’s “Application Programming Interface (API)” for advertising data. The method referred to is the “Ad Account Delivery Estimate”, which is understood as a tool that allows advertisers to manage functionalities related to advertising and ad management published through Facebook. This method enables obtaining various information, including an estimate of “Monthly Active Users” (MAU) on Facebook that usually match specified criteria such as age, gender, and geographic attributes. The data obtained from the API is publicly accessible and can be used by anyone or users with a Facebook advertising account [44].

For this study, the API shows connections of Facebook user accounts that were originally created in Venezuela and are now connecting abroad, in this case Colombia. Facebook API data is collected every 15 days for better tracking of resulting connections. The estimates presented in this document are obtained using specific filters and based on the behavior of Facebook users over the last 30 days.

The data is based on self-reported information provided by each Facebook user, such as their current city, city of origin, and characteristics of their friend network (e.g., having at least two Facebook friends in the home country and two Facebook friends in the destination country) in order to validate the existence of an active account. It is then possible to estimate the number of “migrants” in Colombia by residence and in two time periods, which are compared to validate the percentage variation of some record changes. As mentioned in [45], the documentation of Facebook’s advertising platform defines users who have “lived in country X” as “people who used to live in country X and now live abroad.” Due to the lack of detailed information in this regard, it is unclear what specific criteria Facebook’s advertising platform uses to establish the previous country of residence for each user or the times they lived there. Despite this lack of documentation, it is possible to understand that two factors play a key role in identifying expatriates on Facebook. The first factor is the “current city” and “hometown” self-reported in the “places you’ve lived” list that users or accounts complete for their Facebook profile. The second factor is the friendship structure in the network (for example, having at least two Facebook friends in the home country and two Facebook friends in the destination country [45]).

As for limitations, it is important to note that the estimates presented are not designed to match censuses or other official sources. Facebook does not provide online censuses or statistics on refugees and migrants. Consequently, these estimates should be considered as a source of data that can be used for triangulation and identification of trends, variations in the behavior of accounts originating from Venezuela. Facebook only provides information on certain population groups (in this case, Venezuelan expatriates). Moreover, it does not provide statistical or historical data, so there will be gaps in information in some months or years when no queries were made. These limitations are commonly referenced in studies of this kind, where Facebook API data is used for analysis and estimations of certain populations, as mentioned in [46].

However, when carefully evaluating the validity of Facebook’s estimates and comparing them with data from reliable sources, it is concluded that the estimates can be used for trend analysis and early warning purposes. Additionally, it is a suitable source of data for the study of short-term migration dynamics between Venezuela and Colombia.

With the aim of cross-referencing the data obtained through the specified methodology with official data, the following procedure was conducted. Firstly, the estimated number of Venezuelan migrants at the departmental level was aggregated. Subsequently, for all the departments in the country, the correlation coefficient between the aggregated variable and the official estimates of Venezuelan migrants at the departmental level was calculated. The resulting correlation coefficient was 0.98, which is statistically significant at the 1% level. This finding validates the robustness of the estimated number of Venezuelan migrants in the country using the proposed methodology.

**Fig 2:**
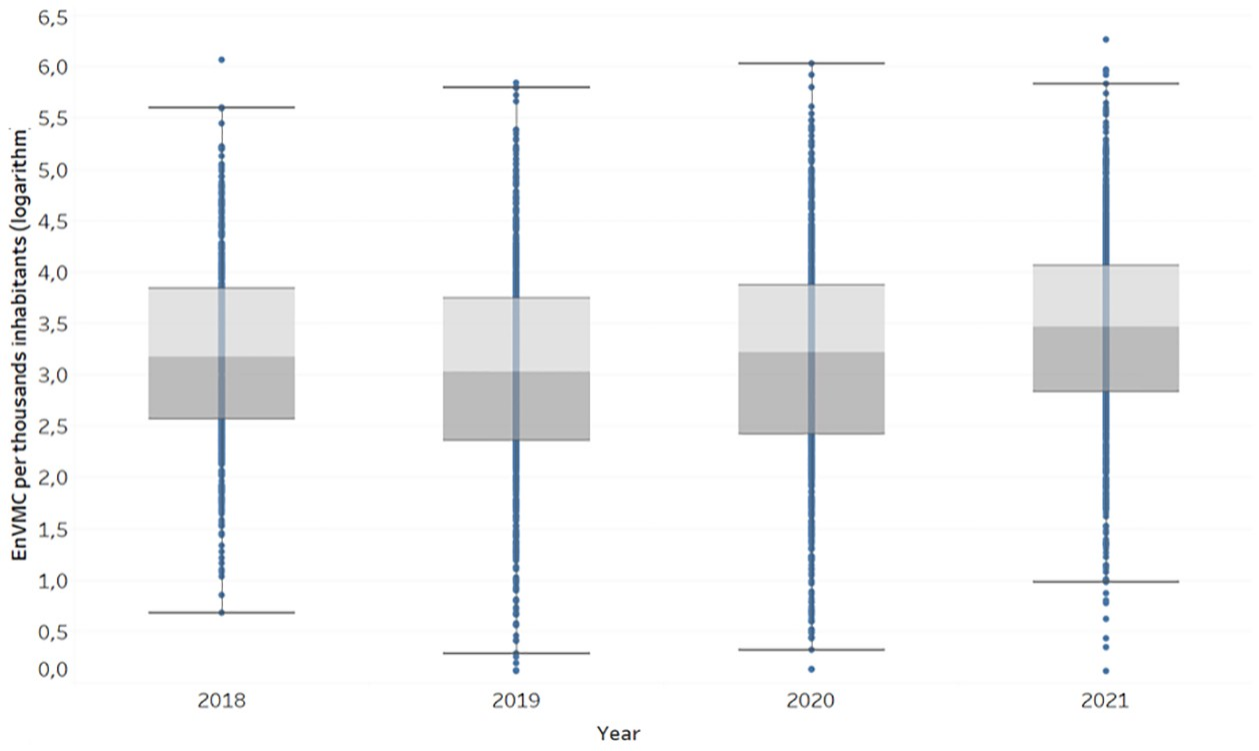
*EnVMC* per thousand inhabitants during the period 2018-2021 (logarithmic scale).

For the purposes of this study, the independent variable of interest is the estimated number of Venezuelan migrant connections in each municipality *i* of Colombia in year *t*: $EnVMC_{t}^{i}$. This variable is a proxy for the number of Venezuelan migrants in the absence of official data at the municipal level. The evolution of *EnVMC* in Colombia during the period of analysis of this research is presented in Fig 2.

**Fig 3:**
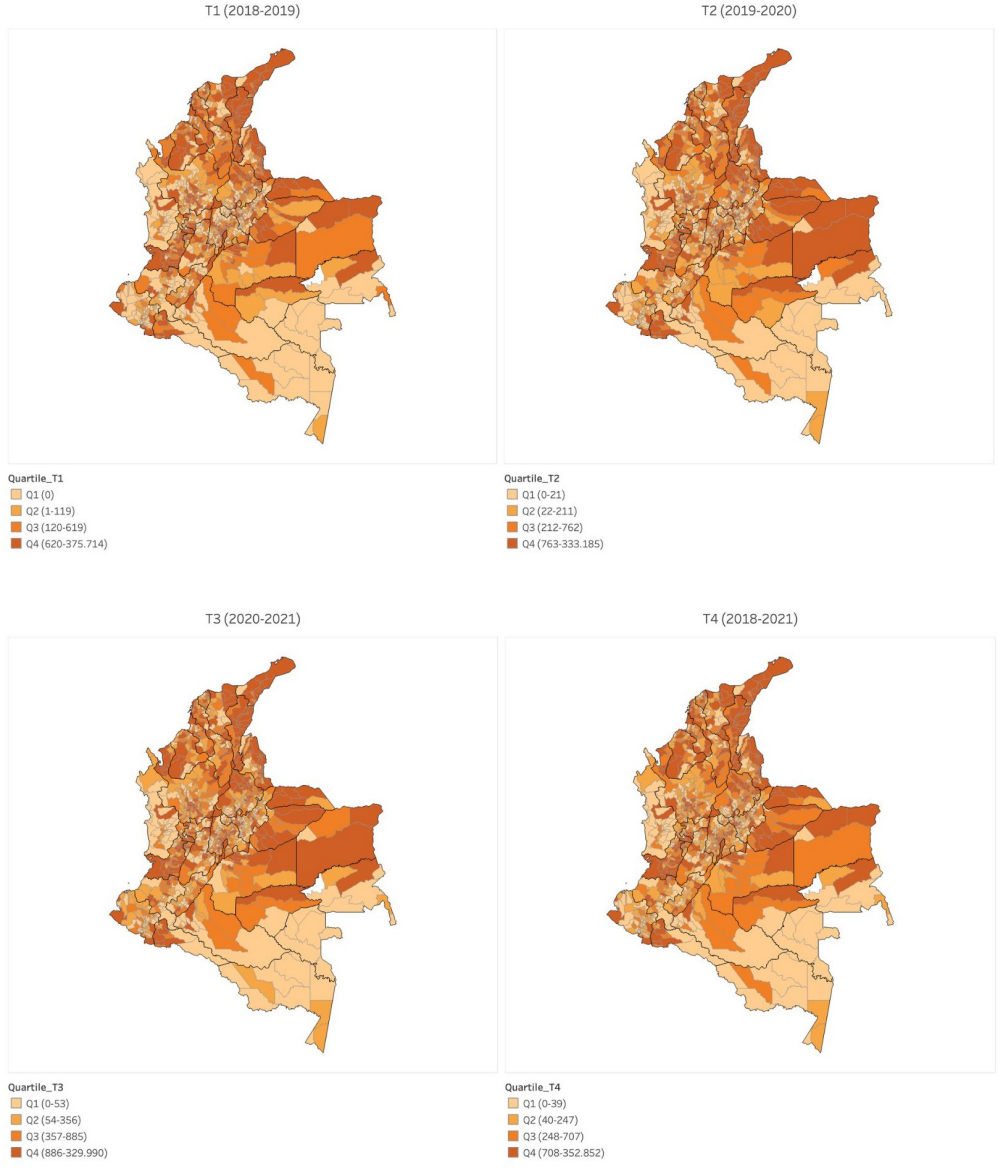
Municipal distribution of the number of Venezuelan migrants in each municipality of Colombia for the initial year of periods *T*_1_ (i.e. 2018-2019) to *T*_4_ (i.e. 2018-2021).

Although the data source provides biweekly measurements of this data, the annual average value of EnVMC in each municipality will be considered as the principal variable. The purpose of this is to prevent economic seasonality and labor commutation rates between municipalities from affecting the estimation of the magnitude of the number of migrants in each municipality. With the aim of presenting an initial approximation to the spatial distribution of the number of migrants in the country, Fig 3 displays the distribution of the number of migrants per municipality in Colombia for the initial year of each of the periods *T*_1_ to *T*_4_.

Fig 3 suggests that there is no discernible pattern of migrant concentration in municipalities near the border within Colombia and Venezuela. However, it is indeed observed that there is a greater concentration of migrants in urban centers that are economic development hubs of the country, such as the cities of Bogota, Medellin, Barranquilla, or Cali. This observation hints that the settlement decisions of Venezuelan migrants are primarily influenced by factors related to the economic characteristics of Colombian municipalities, rather than transportation costs from their country of origin to the receiving municipalities.

To provide a comprehensive analysis of the quantitative relationship between the migration of Venezuelan citizens and urban expansion in Colombia, we consider not only the estimated number of migrants, captured by the variable $EnVMC_{t}^{i}$, but also a variable representing the flow of Venezuelan migrants: Venezuelan Migrant Flow Index *VMF*^*i*^_[*t*,*t*+*k*]_. This variable measures the proportion of population growth between years *t* and *t* + *k* in each municipality that can be explained by the presence of Venezuelan migrants (Eq 2).

As in Eq 1, *i* represents each municipality in the country and *t* corresponds to each year between 2018 and 2020. With the inclusion of the variable *VMF*^*i*^, our research approach focuses not only on how marginal changes in the number of Venezuelans affect the growth of the urban footprint, but also on how the increase in migratory flow, weighted by the total population, influences the phenomenon of urban footprint expansion in the municipalities of Colombia. We define *VMF*^*i*^_[*t*,*t*+*k*]_ as:

$$\begin{array}{c} {VMF^{i}}_{\left[t,t+k\right]}=\frac{{EnVMC^{i}}_{t+k}-{EnVMC^{i}}_{t}}{\text{Total}\;{\text{population}}_{t}^{i}}\cdot 100. \end{array}$$ (2)

It is worth noting that Fig 1 displays a significant shift in Venezuelan migration dynamics during 2020, a year of particular interest due to mobility restrictions implemented in the country as a strategy to mitigate the impact of COVID-19. The above justifies the choice of the four analysis periods specified in the previous section. It is important to note that the estimated number of connections of Venezuelan migrants may be influenced by the internet coverage and connectivity rates in different municipalities of the country. In order to correct for this potential bias, an additional variable was included in the model: municipal internet penetration index (*mipi*^*i*^), which represents the internet coverage at each municipality in Colombia. This allows isolating the effect of interest in this study, as the estimated coefficients for the variables of interest, *EnVMC*^*i*^ and *VMF*^*i*^, will specifically reflect the effect of the estimated number of Venezuelan migrants and not the internet coverage rate.

In order to account for socio-economic factors (in addition to Venezuelan migration) that could influence urban growth, we consider the following variables: the multidimensional poverty index (*imp*^*i*^)-this is a measure of poverty that identifies the proportion of the population that exhibits various deficiencies, classified into five dimensions: educational conditions, conditions of childhood and youth, employment, health, and housing conditions-, the municipal aging index (*mai*^*i*^) -which represents the ratio of people over 60 years old to those under 15 years old-, and the proportion of urban population. Additionally, to analyze the heterogeneous footprint expansion according to the degree of urbanization of municipalities, a dummy variable *city*^*i*^ was included, indicating whether the municipality is a city-urban agglomeration, or a rural municipality. Table 1 presents the descriptive statistics of all variables considered in this study. The source of the control variables used in this research is the DANE.

**Table 1:** Summary statistics of the variables used in this research.

Variable	Obs	Mean	Std.Dev.	Min	Max	Description
*AFEI*_[2018,2019]_	1121	0.009	0.039	0	0.586	Anthropogenic Footprint Expansion Index
*AFEI*_[2019,2020]_	1121	0.002	0.012	0	0.241	
*AFEI*_[2020,2021]_	1121	0.005	0.028	0	0.478	
*AFEI*_[2018,2021]_	1121	0.008	0.041	0	0.62	
*EnVMC*_2018_	960	1470.929	15255.05	0	427143	Estimated number of Venezuelan migrants
*EnVMC*_2019_	960	1492.907	13151.09	0	324286	
*EnVMC*_2020_	960	1857.253	15184.16	0	342083	
*EnVMC*_2021_	960	1688.958	12023.46	0	317897	
*VMF*_[2018,2019]_	960	0.579	3.542	-41.844	33.006	Venezuelan Migrant Flow Index
*VMF*_[2019,2020]_	960	0.615	3.504	-33.111	36.862	
*VMF*_[2020,2021]_	960	1.251	4.158	-38.169	35.873	
*VMF*_[2018,2021]_	960	2.422	4.953	-26.069	44.375	
*mipi*	1117	0.056	0.067	0	0.457	% of pop. with internet access
*imp*	1121	0.418	0.173	0.045	0.985	% of pop. in multidimensional poverty (2018)
*mai*	1121	0.599	0.315	0.048	2.121	Municipal aging index (2018)
% Urban pop_2018_	1121	0.437	0.239	0	0.999	% of the pop. living in urban areas
% Urban pop_2018_	1121	0.44	0.239	0	0.999	
% Urban pop_2018_	1121	0.443	0.24	0	0.999	
*city*	1121	0.428	0.495	0	1	1 = urban agglomeration; 0 = rural

## Statistical model specification

The main objective of the proposed econometric models is to rigorously analyze the quantitative relationship between the independent variables $EnVMC_{t}^{i}$ and *VMF*^*i*^_[*t*,*t*+*k*]_ with the dependent variable *AFEI*^*i*^_[*t*,*t*+*k*]_ during the analysis periods mentioned in the previous section. This approach aims to achieve two objectives. Firstly, it seeks to examine the impact of a marginal increase in the number of Venezuelan migrants during the initial year of the analysis period on urban growth throughout the specified period. Secondly, it aims to investigate the relationship between variations in migrant influx and urban growth. The basic structure of the proposed model is presented in Eq 3.

$$\begin{array}{c} AFEI_{T_{k}}^{i}=\alpha^{i}+\beta \cdot ln\left(EnVMC_{T_{k_{0}}}^{i}\right)+\gamma \cdot \left(VMF_{T_{k_{0}}}^{i}\right)+\theta \cdot \left(SD_{T_{0}}^{\prime i}\right)+\varepsilon_{T_{k}}^{i}, \end{array}$$ (3)

where *T*_*k*_ is each of the period analysis from *T*_1_ to *T*_4_. $AFEI_{T_{k}}^{i}$ represents the Anthropogenic Footprint Expansion Index in municipality *i* during period *T*_*k*_, *α*^*i*^ represents the fixed effects of the department to which each municipality belongs, allowing to control the heterogeneities that characterize each department of the country, $ln(EnVMC_{T_{k_{0}}}^{i})$ represents the natural logarithm of the number of Venezuelan migrants in municipality *i* during the initial year of period *T*_*k*_ and $VMF_{T_{k_{0}}}^{i}$ is the Venezuelan Migrant Flow Index during the period *T*_*k*_.

Complementary, $SD_{T_{0}}^{\prime i}$ is a vector of sociodemographic variables, namely: the Incidence of multidimensional poverty (*imp*^*i*^), Municipal aging index (*mai*^*i*^), the percentage of urban proportion in $T_{k_{0}}$ (*UrbanPop*), *city*, a dummy variable indicating whether the municipality in question is a city-urban agglomeration or a rural municipality, and *mipi*^*i*^ is the municipal internet penetration index.

The statistical analysis was conducted in three stages using cross-sectional data for each of the analysis periods *T*_1_ to *T*_4_. At the first stage of the analysis, we ran ordinary least squares regression (OLS) to study the quantitative relationship between Venezuelan migration and urban expansion in Colombia. At the second and third stage of the quantitative research we ran, respectively, a Durbin spatial error model and a Durbin model for spatial lags, in order to investigate whether there is a spatial relationship between the variables of interest. All econometric analysis was conducted using STATA 16 software. In the following section we present the results of the estimated models.

## Results

Variable *EnVMC* exhibits a positive and statistically significant effect on urban expansion during the periods *T*_1_ and *T*_2_. Results from Table 2 indicate that a 1% increase in the number of Venezuelan migrants in 2018 leads to a 0.0008-point increase in the urban expansion index for the period *T*_1_, which is statistically significant at the 5% level. A similar interpretation applies to period *T*_2_, although the magnitude of the independent variable’s marginal effect is four times smaller. Results for *T*_3_ and *T*_4_ are not statistically significant. These findings suggest that, after the COVID-19 pandemic, the effect of Venezuelan migration on the urban expansion in Colombia diminished significantly. The explanation for this finding lies in mobility restrictions, economic disruptions caused by the pandemic, and political changes in the Colombia-Venezuela relationship between 2018 and 2021.

**Table 2:** OLS robust regression analysis on the Anthropogenic Footprint Expansion index.

	OLS Robust regression model			
Independent variables	2018-2019	2019-2020	2020-2021	2018-2021
	T1	T2	T3	T4
*ln*(*EnVMC*)	0.008**	0.002**	0.003	0.005
	-0.003	-0.001	-0.002	-0.004
*VMF*	0.001*	0.000	0.000	0.000
	0.000	0.000	0.000	0.000
% urban population	-0.036***	-0.006*	-0.017**	-0.037***
	-0.012	-0.003	-0.008	-0.014
*imp*	-0.051***	-0.002	-0.036***	-0.061***
	-0.015	-0.006	-0.011	-0.018
*mai*	-0.009	-0.005*	-0.013**	-0.045***
	-0.014	-0.003	-0.005	-0.011
*city*	0.022***	0.004**	0.014***	0.018***
	-0.007	-0.002	-0.004	-0.007
*mipi*	0.239***	0.055***	0.106***	0.227***
	-0.067	-0.014	-0.032	-0.066
Constant	-0.014	-0.013*	0.001	0.006
	-0.024	-0.008	-0.015	-0.026
Observations	522	714	783	522
R-squared	0.411	0.234	0.31	0.406
AIC	-1755.162	-4050.119	-3326.604	-1704.602
BIC	-1606.144	-3880.996	-3154.068	-1555.584
Log-likelihood	912.5812	2062.06	1700.302	887.301

Robust standard errors in parentheses

*** p<0.01,

** p<0.05,

* p<0.1

AIC: Akaike Information Criterion. BIC: Bayesian information criterion.

With respect to the flow of migrants, a marginal increase in this indicator, represented in the variable *VMF* implies an increase of 0.001-units in the urban expansion index *AFEI* during period *T*_1_, and this effect is statistically significant. However, this effect vanishes in subsequent periods. This finding, combined with the previous paragraph’s insights, indicate that migration had a significant impact on short-term urban expansion previous to year 2020, but that gradually faded in subsequent years.

A notable result is that the multidimensional poverty index (*imp*) has a negative and statistically significant effect on urban expansion in Colombia. In other words, poverty is inversely related with urban growth in Colombia. For the *T*_1_ period, a marginal increase in *imp* leads to a 0.051-point decrease in the urban expansion index. Similar to the aforementioned variables, this effect diminishes over time. Nevertheless, the relationship of variables *imp* and urban expansion is of greater magnitude and statistical significance in period *T*_4_: an increase in *imp* leads to a 0.061-point decrease in variable *AFEI*. This means that, in the medium term, poverty is a crucial variable that negatively affects urban growth.

An analogous pattern emerges concerning variable *mai*, exhibiting a negative and statistically significant effect in periods *T*_2_ to *T*_4_, with the most pronounced effect observed in the last period: a one-unit increase in the *mai* index results in a 0.045-point decrease in *AFEI* variable. This finding highlights that the effect of a higher presence of young people on urban growth is predominantly a medium-term phenomenon rather than a short-term one.

The variable representing the proportion of urban population exhibit a consistent and statistically significant negative effect on the *AFEI* index in the presented model. This finding indicates that in Colombia, cities with a smaller urban population are witnessing more significant growth in the short run. This observation may be attributed to the economic dynamics of intermediate cities. It is important to highlight that in [30], the authors identify a positive and statistically significant impact of the same variable on urban expansion. This disparity can be attributed to the variances in the time frames examined in the respective studies, with the previous research considering a medium-term perspective while the current study focuses on the short term.

Lastly, city variable shows a positive and significant effect on the *AFEI* index, indicating that cities, as opposed to rural areas, are the municipalities experiencing expansion. The internet penetration rate (*mipi* variable) exhibits a similar behavior. This finding should be further explored in future research, which could incorporate internal migration and study rural-urban migration patterns as explanatory factors for city growth in Colombia.

It is worth noting that the econometric models presented in this study employed the variance inflation factor as a tool to assess potential multicollinearity among the variables. This test confirmed the absence of multicollinearity among the variables used. With the aim of studying local patterns of spatial autocorrelation and identifying regions that have experienced a greater increase in the dependent variable of interest in the econometric model (*AFEI* variable), the Local Moran’s Spatial Autocorrelation Index was estimated. This indicator reveals that the metropolitan areas of the cities of Bogotá, Medellin, and Barranquilla have witnessed the most significant growth in urban footprint during the analysis period. These cities, in fact, are where the highest number of Venezuelan migrants arrived in Colombia, according to the *EnVMC* variable.

In order to assess the presence of spatial autocorrelation in the statistical model under consideration, i.e., whether the phenomenon of urban footprint expansion in Colombian municipalities and its relationship with the arrival of Venezuelan migrants is geographically clustered, the Moran I-Index was calculated for the model specified in Eq 3 for all analysis periods in the study. The Moran I-Index yielded positive values of 16.7, 7.17, 14.01, and 19.69 for periods *T*_1_, *T*_2_, *T*_3_, and *T*_4_, respectively. For all these periods, the p-value was less than 0.001, indicating that, with a statistical significance level below 1%, the studied phenomenon exhibits geographic clustering. This implies a significant geographical effect, that is, nearby locations exhibit similar dynamics, and therefore justifies the utilization of a spatial error model. The Lagrange multiplier also exhibits a p-value less than 0.001, providing evidence of a spatial lag and further validating the adoption of a spatial lag model. The outcomes of both the spatial error model and the spatial lag model are presented in Tables 3 and 4, respectively.

**Table 3:** Spatial error regression on the Anthropogenic Footprint Expansion Index.

	Spatial error regression			
Independent variables	2018-2019	2019-2020	2020-2021	2018-2021
	T1	T2	T3	T4
*ln*(*EnVMC*)	0.008***	0.002***	0.003***	0.005**
	-0.002	-0.001	-0.001	-0.002
*VMF*	0.001	0.000	0.000	0.000
	-0.001	0.000	0.000	0.000
% urban population	-0.029**	-0.006*	-0.016**	-0.033***
	-0.012	-0.003	-0.007	-0.013
*imp*	-0.042*	-0.002	-0.034***	-0.052**
	-0.022	-0.006	-0.012	-0.023
*mai*	-0.014	-0.005*	-0.011**	-0.040***
	-0.011	-0.003	-0.005	-0.012
*city*	0.019***	0.003	0.012***	0.015**
	-0.006	-0.002	-0.004	-0.007
*mipi*	0.211***	0.054***	0.104***	0.216***
	-0.042	-0.012	-0.023	-0.044
*lambda*	0.938***	0.659***	0.898***	0.950***
	-0.061	-0.295	-0.1	-0.05
Constant	0.039	-0.01	0.071**	0.066
	-0.056	-0.015	-0.032	-0.06
Observations	522	714	783	522
AIC	-1770.131	-4045.005	-3334.24	-1726.255
BIC	-1599.824	-3857.599	-3143.051	-1555.948
Log-likelihood	925.0656	2063.502	1708.12	903.1273

Standard errors in parentheses

*** p<0.01,

** p<0.05,

* p<0.1

AIC: Akaike Information Criterion. BIC: Bayesian information criterion.

The results presented indicate that the Log-likelihood statistic consistently yields higher values in the spatial lag model compared to the OLS and spatial error models. Consequently, this approach is considered to have the highest statistical explanatory power. In general terms the results presented in Tables 3 and 4 are consistent with those of the OLS model (Table 2).

**Table 4:** Spatial lag regression on the Anthropogenic Footprint Expansion Index.

	Spatial lag regression			
Independent variables	2018-2019	2019-2020	2020-2021	2018-2021
	T1	T2	T3	T4
*ln*(*EnVMC*)	0.008***	0.002***	0.003***	0.005**
	(0.002)	(0.001)	(0.001)	(0.002)
*VMF*	0.001	-0.000	0.000	-0.000
	(0.001)	(0.000)	(0.000)	(0.000)
% urban population	-0.028**	-0.006*	-0.015**	-0.033***
	(0.012)	(0.003)	(0.006)	(0.012)
*imp*	-0.037*	-0.000	-0.031***	-0.048**
	(0.021)	(0.006)	(0.011)	(0.022)
*mai*	-0.013	-0.005*	-0.011**	-0.040***
	(0.010)	(0.003)	(0.005)	(0.011)
*city*	0.019***	0.003	0.011***	0.014**
	(0.006)	(0.002)	(0.004)	(0.007)
*mipi*	0.216***	0.054***	0.105***	0.219***
	(0.041)	(0.012)	(0.023)	(0.043)
*rho*	0.960***	0.800***	0.929***	0.965***
	(0.039)	(0.178)	(0.070)	(0.035)
Constant	0.034	-0.014	0.059**	0.059
	(0.043)	(0.015)	(0.030)	(0.045)
Observations	522	714	783	522
AIC	-1796.357	-4050.636	-3345.59	-1751.678
BIC	-1626.051	-3863.23	-3154.401	-1581.371
Log-likelihood	938.1787	2066.318	1713.795	915.839

Standard errors in parentheses

*** p<0.01,

** p<0.05,

* p<0.1

AIC: Akaike Information Criterion. BIC: Bayesian information criterion.

It is worth noting some differences in the statistical significance of the independent variables. The main difference corresponds to the statistical significance of the variable *EnVMC*. Both in the spatial error regression model and in the spatial lag regression model, this variable is statistically significant for all analysis periods. However, there is evidence of a decrease in the magnitude of the effect, which is more marked for period *T*_1_ than for the other periods (Tables 3 and 4).

## Additional statistical tests

With the aim of further exploring the statistical relationship between Venezuelan migration and urban growth, various complementary statistical tests were conducted. Initially, the hypothesis was proposed that Venezuelan migration would have a greater impact on Colombian municipalities close to the border crossings between Colombia and Venezuela. It was assumed that transportation costs could act as a barrier to Venezuelan mobility within Colombia. To address this issue, a variable was created to identify the distance of each municipality in the country to the nearest migratory border crossing point. Subsequently, all municipalities were classified into four groups based on quartiles of the mentioned variable. Regression models, as presented in Table 2, were then run for each quartile.

However, the results of this analysis are inconclusive, as no significant relationship was found between the impact of the number of Venezuelan migrants (variable *EnVMC*) on the urban expansion of Colombian municipalities and the distance to migratory crossing points. Nevertheless, it is necessary to emphasize that some unobservable variables may be influencing this phenomenon, which is not being reflected in the statistical model we are proposing in the present research. Similarly, there might be an effect among the mentioned variables, but it may only be manifested over longer periods of time. This phenomenon is beyond the scope of our current investigation and should be addressed by future research efforts.

Our hypothesis is that Venezuelans would be attracted to settle in municipalities with a more dynamic economy, and consequently, the effects of migration on urban expansion would be felt more strongly in those municipalities whose production of goods and services contributes more significantly to the national value added, because these municipalities can offer greater employment opportunities and more development prospects. To assess this idea, all municipalities were divided into four groups based on their value added. Subsequently, the corresponding regression model was performed within each group.

**Fig 4:**
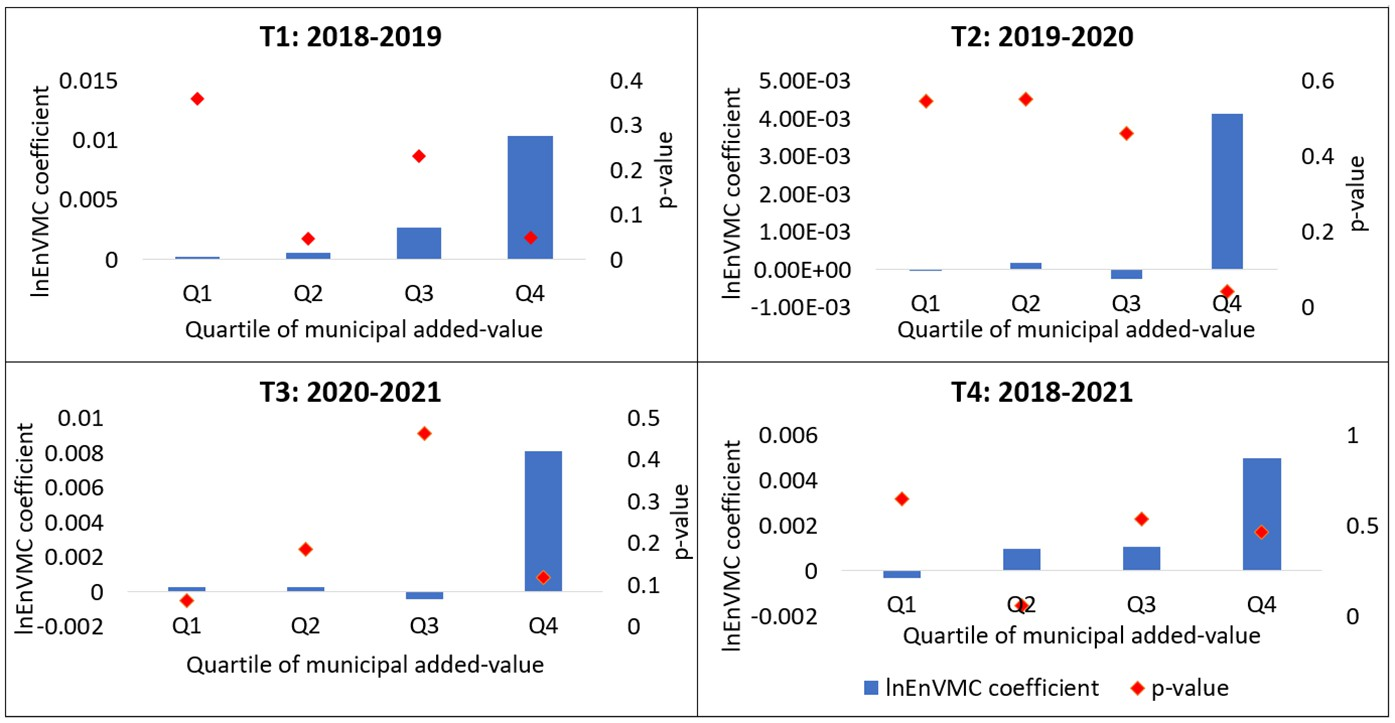
Magnitude of the coefficient of variable *EnVMC* and its p-value in the regression within each quartile of municipalities according to added value.

The results of this second analysis were significant. During the study periods, it was observed that Venezuelan migration (measured by the coefficient of the variable *EnVMC*) had a more prominent effect in municipalities that contributed more to the country’s value added. Additionally, these municipalities presented the lowest p-value (see Fig 4). The results of these statistical tests suggest that Venezuelan migration in Colombia is more influenced by the level of economic development of municipalities than by the distance to border crossings.

Previous studies (e.g., [30]) have shown that the armed conflict in Colombia has had a significant impact on patterns of internal migration and the growth of urban centers. In order to test this hypothesis within the context of the current research, new econometric regressions of the model proposed in this study were conducted, incorporating the incidence of armed conflict index (IIRCA) as an independent variable. This index is a standardized numerical measure that scores municipalities in the country based on the extent to which they have been affected by the armed conflict in Colombia [47]. The IIRCA includes variables such as the number of armed actions in the municipality, homicide rate, kidnapping rate, coca cultivation rate, and forced displacement, among others. When running the regression models, given the statistical methodology proposed in this research we observed that this variable does not have a statistically significant effect on the urban expansion index in the country. However, it is crucial that subsequent research endeavors encompass this phenomenon. We conclude that the impact of the armed conflict on urban expansion in Colombia is a medium and long-term phenomenon, rather than a short-term one.

## Discussion

Overall, this research emphasizes the effect of Venezuelan migration on short-term urban expansion in Colombia, both before and after the COVID-19 pandemic, showing that before the pandemic Venezuelan migration had a significant effect on urban sprawl, but that this faded after the pandemic. The effect of migration on urban growth is predominantly a short-term phenomenon rather than a medium-term one. In the latter, factors such as incidence of multidimensional poverty, or demographic structure of population become relevant. This is evident in the negative and statistically significant impact of the aging index on urban growth: the higher the ratio between the number of older adults and the number of young individuals, the lower the rate of city expansion.

There is evidence of spatial autocorrelation in the studied phenomenon. This means that, in the context of the studied phenomenon, geographically close municipalities exhibit similar dynamics. This justifies the use of spatial statistics methods, as presented in this work. The implementation of spatial econometrics models corroborates the results described in the previous paragraph, validating the robustness of the findings presented throughout this research.

With the aim of testing the hypothesis that the impact of Venezuelan migration on urban expansion is more pronounced in municipalities proximate to the border crossings between Colombia and Venezuela, we conducted additional econometric tests. These tests aimed to assess the significance and magnitude of migration, segmenting the national territory based on the distance to the border crossings between Colombia and Venezuela. However, the findings are not significant. It is concluded that transportation costs are not a determining factor in the decisions of where Venezuelan migrants locate. On the contrary, the effect of Venezuelan migration is bigger in more developed municipalities (municipalities that most contribute to the national value added). This is an indication that the workforce of Venezuelan migrants has more incentives to settle in population centers with a higher degree of economic development.

Finally, urban growth depends not only on international migration phenomena but also on endogenous population growth and internal migration flows. In fact, because of the armed conflict and the economic disparity between urban and rural areas in Colombia, there has been a pronounced population movement from the national peripheries to large cities [28, 30]. While this study captures endogenous population growth through the controls, again, due to the precariousness of the data, it is difficult to capture the effect of internal migration on urban growth.

One of the study’s main limitations is the source of the Facebook connections data. Since these data come from Venezuelan accounts connecting in Colombia, there may be a bias regarding the official data reported by the national government. For example, not all Venezuelan refugees and migrants likely have Facebook accounts, which would underestimate the reporting of migrants in the country. On the other hand, those with an active account may have a different socioeconomic profile than most of the Venezuelan population. Thus, Facebook connections may differ in age groups from the total universe of Venezuelan migrants in the country.

Future research should investigate the indirect impact of Venezuelan migration on urban growth, specifically how the age composition of the migrant population affects the aging index of municipalities and, in turn, urban expansion. The currently available data does not allow for this type of analysis to be conducted. Subsequent studies should also explore how patterns of rural-urban internal migration affect city growth in the medium and long term. It is also important for future research to assess the impact of Venezuelan migration on urban expansion considering more complex units of analysis than municipal boundaries, such as regions defined by the functional relationships established at a supramunicipal level in the country.
